# Characterization of the *PMT* Gene Family in *Cryptococcus neoformans*

**DOI:** 10.1371/journal.pone.0006321

**Published:** 2009-07-27

**Authors:** Sven D. Willger, Joachim F. Ernst, J. Andrew Alspaugh, Klaus B. Lengeler

**Affiliations:** 1 Institut für Mikrobiologie, Molekulare Mykologie, Heinrich-Heine-Universität, Düsseldorf, Germany; 2 Department of Veterinary Molecular Biology, Montana State University, Bozeman, Montana, United States of America; 3 Departments of Medicine and Molecular Genetics/Microbiology, Duke University Medical Center, Durham, North Carolina, United States of America; Texas A & M University, United States of America

## Abstract

**Background:**

Protein-*O-*mannosyltransferases (Pmt's) catalyze the initial step of protein-*O-*glycosylation, the addition of mannose residues to serine or threonine residues of target proteins.

**Methodology/Principal Findings:**

Based on protein similarities, this highly conserved protein family can be divided into three subfamilies: the Pmt1 sub-family, the Pmt2 sub-family and the Pmt4 sub-family. In contrast to *Saccharomyces cerevisiae* and *Candida albicans*, but similar to filamentous fungi, three putative *PMT* genes (*PMT1*, *PMT2*, and *PMT4*) were identified in the genome of the human fungal pathogen *Cryptococcus neoformans*. Similar to *Schizosaccharomyces pombe* and *C. albicans*, *C. neoformans PMT2* is an essential gene. In contrast, the *pmt1* and *pmt4* single mutants are viable; however, the *pmt1/pmt4* deletions are synthetically lethal. Mutation of *PMT1* and *PMT4* resulted in distinct defects in cell morphology and cell integrity. The *pmt1* mutant was more susceptible to SDS medium than wild-type strains and the mutant cells were enlarged. The *pmt4* mutant grew poorly on high salt medium and demonstrated abnormal septum formation and defects in cell separation. Interestingly, the *pmt1* and *pmt4* mutants demonstrated variety-specific differences in the levels of susceptibility to osmotic and cell wall stress. Delayed melanin production in the *pmt4* mutant was the only alteration of classical virulence-associated phenotypes. However, the *pmt1* and *pmt4* mutants showed attenuated virulence in a murine inhalation model of cryptococcosis.

**Conclusion/Significance:**

These findings suggest that *C. neoformans* protein-*O-*mannosyltransferases play a crucial role in maintaining cell morphology, and that reduced protein-*O-*glycosylation leads to alterations in stress resistance, cell wall composition, cell integrity, and survival within the host.

## Introduction

Protein-*O-*glycosylation is an essential, evolutionary conserved protein modification that has been studied extensively in the yeasts *Saccharomyces cerevisiae* and *Candida albicans*. This process has also been identified in other fungal species [1]–[3], in higher eukaryotes [4]–[6] and in certain bacterial genera [7], [8]. In yeasts and fungi, protein-*O*-glycosylation is initiated at the luminal side of the endoplasmic reticulum (ER) by the addition of a mannosyl residue to specific serine/threonine residues of proteins entering the secretory pathway [9], [10]. This first modification is derived from the polyisoprenoid carrier lipid dolichyl phosphate-activated mannose (Dol-P-Man), followed by the addition of short, linear, mannosyl-rich glycans. Maturation and further modification of the glycosyl chains occur in the Golgi apparatus.

The initial step of *O-*glycosylation is catalysed by a highly conserved family of integral ER membrane proteins, the protein-*O-*mannosyltransferases (Pmt's) [11]. The Pmt family was initially identified in *S. cerevisiae*, in which seven *PMT* genes have been identified [3], [11]. Broader phylogenetic analyses reveal that protein*-O-*mannosyltransferases can be grouped into three major subfamilies, corresponding to *S. cerevisiae* Pmt1p, Pmt2p and Pmt4p. Many fungal species contain only three *PMT* genes in their genome, one for each subfamily.

In *S. cerevisiae*, *O-*glycosylation affects the stability, localization, and function of proteins, preventing the exportation of misfolded proteins from the ER [12]–[14]. Similarly, in the human pathogenic fungus *C. albicans*, correct *O-*mannosylation is important for morphogenesis, adherence to host cells, and virulence [15]. *C. albicans* proteins that are modified by Pmt's include chitinases, proteases, proteins involved in glucan synthesis, heat-shock proteins, and cell-surface antigens important for virulence (reviewed in [2], [3]). In addition to secreted proteins, the proper function of various receptors requires intact protein-*O-*glycosylation activity [14], [16].

Although protein-*O-*mannosylation seems to be less abundant in higher eukaryotes, defects in this process result in human disease, such as muscle-eye-brain disease (MEB) and Walker–Warburg Syndrome (WWS) [17]. Furthermore, a targeted deletion of POMT1, which causes WWS in mice resulted in embryonic lethality [18]. Mutation of *Drosophila PMT* homologs alters muscle structures and the alignment of adult cuticle [19], [20]. Therefore, the analysis of the *PMT*-gene family in different species over the last few years revealed that protein-*O-*mannosylation activity is involved in central developmental and growth processes in both uni- and multicellular eukaryotes.

*C. neoformans* is an opportunistic human fungal pathogen causing life-threatening meningoencephalitis. This fungus produces several extracellular factors that are important for virulence, including an extensive polysaccharide capsule, several secreted hydrolytic enzymes, and the cell wall-targeted pigment melanin (reviewed in [21]). Since protein-*O-*glycosylation predominantly affects extracellular proteins, any defect in this biological process may affect the interface of pathogenic microorganism and the host.

*PMT4*, one of three putative Pmt orthologs in the basidiomycete *C. neoformans*, has recently been identified. Disruption of the *C. neoformans PMT4* gene results in dramatic effects on virulence [22]. In addition, *pmt4* mutant strains show morphological defects and alterations of the cell wall, possibly due to changes in glycan composition/synthesis. However, the relative roles of *C. neoformans PMT1* and *PMT2* have not yet been elucidated. In this paper we report a continuing analysis of the complete *C. neoformans PMT* gene family in two biologically distinct varieties, var. *grubii* and var. *neoformans*. We identified three *C. neoformans PMT* genes, and we have begun to define their overlapping and distinct functions in stress response, cell wall integrity, and survival in the host.

## Results

### Three *PMT* genes are present in *C. neoformans*

Fungal genomes typically contain multiple genes encoding protein-*O-*mannosyltransferases. For example, *S. cerevisiae* contains seven *PMT* genes (*PMT1-7*), while five *PMT* genes are present in the genome of the human pathogenic fungus *C. albicans*. Three putative *PMT* genes were recently identified in the basidiomycetous yeast *C. neoformans* variety *neoformans* (serotype D), one for each major sub-family: *PMT1* (CND06150 on chromosome 4), *PMT2* (CNJ01930 on chromosome 10), and *PMT4* (CND01240 on chromosome 4) [23]. *C. neoformans* Pmt1 shares 41% amino acid identity and 59% similarity to *S. cerevisiae* Pmt1; *Cn*Pmt2 has 47% identity and 65% similarity to *Sc*Pmt2; and *Cn*Pmt4 has 42% identity and 61% similarity to *Sc*Pmt4. Beyond direct amino acid sequence comparison, a more detailed phylogenetic sequence analysis places each of the three *C. neoformans PMT* genes into one of the three major *PMT* gene families. Hydrophobicity analysis of the predicted Pmt protein sequences revealed a seven-transmembrane helical structure commonly predicted for this class of proteins [9].

Although closely related, *C. neoformans* var. *neoformans* and var. *grubii* strains (serotypes D and A, respectively) have distinct characteristics in terms of intracellular signaling and cellular physiology [24]–[26]. As scientific models, var. *grubii* strains have been used most extensively in pathogenesis experiments, and var. *neoformans* strains have more tractable mating and genetic systems. The three *PMT* genes are also present in the var. *grubii* genome, encoding proteins that are 97–98% identical at the amino acid level to those from var. *neoformans*. For clarity, we have chosen to refer to the var. *grubii* (serotype A) *PMT* genes as *PMT1*A, *PMT2*A, and *PMT4*A; and the var. *neoformans* (serotype D) genes as *PMT1*D, *PMT2*D, and *PMT4*D.

### *PMT* genes of *C. neoformans* are constitutively expressed under various growth conditions

We characterized the transcriptional regulation of the entire *C. neoformans PMT* gene family under various physiologically relevant growth conditions. The wild-type strains JEC21 (serotype D) and H99 (serotype A) were incubated to mid-logarithmic phase in rich medium (YPD) at 30°C and 37°C, salt stress (YPD+1 M NaCl), and capsule-inducing conditions (DMEM). Northern blots and quantitative real-time PCR demonstrated little variation in the expression of any of the three *PMT* genes in either strain variety under these conditions (data not shown). Therefore, the three *C. neoformans PMT* genes are constitutively expressed under most growth conditions. Our data support and confirm the recent observation that the *PMT4*A gene is not induced by changes in temperature or nutrient availability [22].

### Protein-*O-*glycosylation is essential in *C. neoformans*

We used targeted gene disruption to further characterise the biological functions of the *C. neoformans PMT* genes in both serotype backgrounds. Since it has been shown in other organisms that *pmt* mutant strains can be highly sensitive to cell wall destabilizing drugs and drugs targeting protein synthesis, including those used in *C. neoformans* as dominant selectable markers (such as hygromycin B), we used the *ADE2* and *URA5* genes as selectable markers to perform these experiments. The selectable marker cassettes were inserted into the loop five region of the *PMT* genes, which is predicted to be essential for enzyme function [27].

While *pmt1* and *pmt4* single mutant strains could easily be isolated for both serotypes, we were not able to isolate *pmt2* mutant strains from either serotype, even after several rounds of transformation (>300 transformants). This finding suggested that *PMT2* might be an essential gene in *C. neoformans*, as it is in *S. pombe* and *C. albicans* [15], [28]. To further confirm this hypothesis, we isolated a homozygous *ade2/ade2* diploid strain from a cross of strains JEC156 (*MATa ade2 ura5*) and JEC157 (*MATalpha ade2 ura5 lys1*) according to the methods of Sia et al. [29]. By transforming a *pmt2::ADE2* disruption construct into this diploid strain, we were able to isolate heterozygous *PMT2/pmt2::ADE2* strains at similar frequencies (∼10%) compared to the *pmt1* and *pmt4* single mutants. We then allowed three independent heterozygous diploid mutants to sporulate. From this sporulation, we isolated 75 *ADE*+haploid progeny, and all had a wild-type *PMT2* locus. In contrast, the other genetic markers (mating type, *LYS1*) demonstrated expected levels of recombination. We assume that the *ADE*+progeny that are not *pmt2*, either have an ectopic integration of *pmt2::ADE* or have a recombination that restores the *ade2* allele back to wild-type. To determine that the isolated 75 *ADE*+haploid progeny had a wild-type *PMT2* locus we first performed Southern Blot analyses on the diploid strains that were sporulated showing that the *PMT2* and *pmt2::ADE2* alleles were present (data not shown). The progeny were subsequently also analyzed by colony PCR for the nature of the *PMT2* allele present in the strains amplifying a short fragment spanning the region where the *ADE2* marker was integrated (data not shown). The failure to isolate haploid *pmt2* mutants from the sporulation of the *PMT2/pmt2* diploid strongly suggests that the *PMT2* gene is essential.

**Figure 1:**
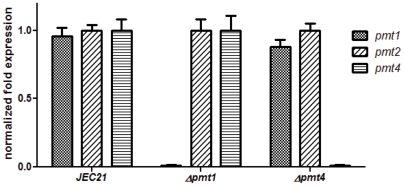
*PMT*s are not differentially expressed and the loss of one *pmt* gene is not compensated by overexpression of another *PMT* gene. The wild-type (JEC21), *Δpmt1* and *Δpmt4* mutant strains were incubated to mid-logarithmic phase in YPD medium and total RNA was isolated. Quantitative real-time PCR was performed on the corresponding cDNA samples to assess relative *PMT1, PMT2*, and *PMT4* transcript abundance compared to the wild-type strain±one standard deviation. The expression values in all three strains are normalized to the *PMT2* expression.

To verify that the insertion of the selectable marker cassette resulted in a loss of the transcript for the *pmt1* and *pmt 4* mutants we performed real time PCRs with *PMT1, PMT2, and PMT4* specific primers on cDNA derived from the serotype D mutants grown at 30°C. As a control we used cDNA derived from the serotype D wild-type strain JEC21. Since we couldn't generate a *pmt2* mutant we normalized the data for each strain to the *PMT2* expression. The results confirm that the *PMT* genes are constitutively expressed with only little to no variation in the wild-type strain JEC21 (Figure 1). In the insertion mutants the transcript of either *PMT1* or *PMT4* is completely abolished in the respective mutant. The real time PCR data also clearly show that none of the *PMT* genes is differentially expressed in any of the mutants to compensate the loss of another *PMT* gene (Figure 1).

### The “Pmt holoenzyme” functions as a dimer or multimer of the individual Pmt proteins

**Figure 2:**
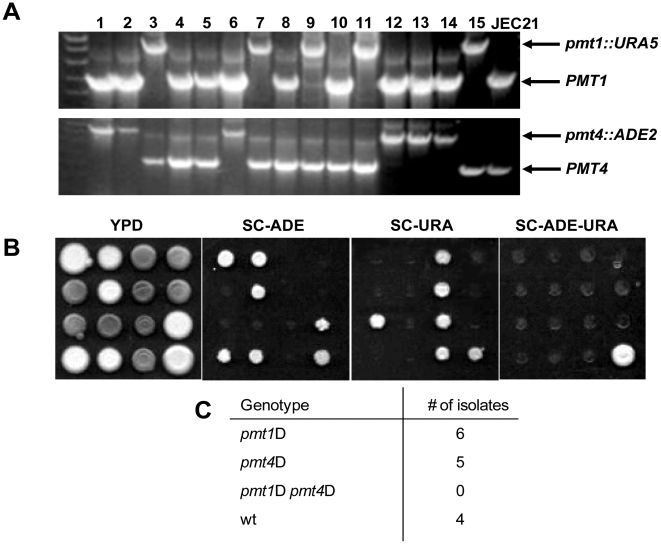
A *pmt1 pmt4* double deletion is lethal in serotype D. Serotype D strains *pmt1*D (*MATa pmt1D*::*URA5 ade2-27*) and *pmt4*D (*MATα pmt4D::ADE2 ura5*) were crossed on standard V8 mating media and individual spores were isolated after several days by micromanipulation. A: 15 individual progeny were analyzed by colony PCR for the presence of the *PMT1* (upper panel) and *PMT4* alleles (lower panel). Position of the respective wild-type and disruption alleles are indicated at right. Wild-type strain JEC21 was used as a control. B: The 15 strains from A were spotted onto the indicated plates, grown for 2–3 days at 30°C and subsequently analyzed for auxotrophic marker distribution. Strains were spotted from top left (#1) to bottom right (wild-type control). C: Summary of the genotypes identified in A and B with respect to *PMT* alleles and mating-type. Mating-type of the individual spores were determined by standard mating reactions using wild-type strains JEC20 (*MATa*) and JEC21 (*MATα*) as tester strains.

It has been shown for other yeasts that Pmt2 forms a heterodimer with Pmt1 proteins and that these dimers show a high protein-*O-*mannosylation activity [28], [30]. Based on this finding and interaction studies of Pmts in higher eukaryotes, i.e. in human, where POMT1 (member of the PMT4 family) and POMT2 (member of the PMT2 family) form heterodimers [31], we predicted that in *Cryptococcus* Pmt2 also forms heterodimers with other Pmts. This model of protein function would predict that the *pmt1 pmt4* double mutation would be synthetically lethal since there would be no other Pmt protein to interact with Pmt2. To test this hypothesis, we crossed the *MAT*alpha *pmt1*D strain with the *MAT*a *pmt4*D strain and isolated individual basidiospores by microdissection. We tested the resulting progeny strains for the following genetic markers: mating type, *PMT1*, *PMT4*, *ADE2*, and *URA5*. The marker segregation pattern demonstrated that the isolated spores were the result of meiotic recombination (Figure 2). More importantly, among the 15 dissected spores we were not able to identify a *pmt1 pmt4* double mutant, strongly suggesting that the *pmt1* and *pmt4* mutations are synthetically lethal. These results suggest that protein-*O-*glycosylation is an essential process in *C. neoformans*, and that either Pmt1 and Pmt4 both interact with Pmt2 to provide the cell with optimal protein-*O-*mannosylation activity or that Pmt1 and Pmt4 have a common essential target independent of Pmt2.

### pmt1 and pmt4 mutant strains show altered cell morphology

**Figure 3:**
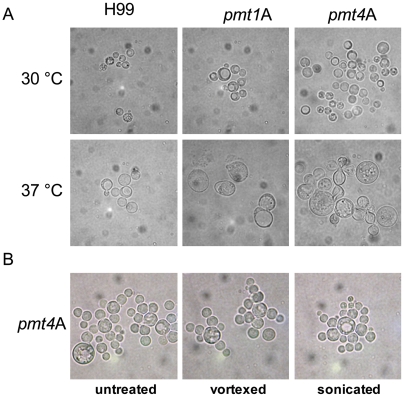
The *pmt1* and *pmt4* disruption strains show altered cell morphology. A: Serotype A wild-type strain H99 and mutant strains *pmt1*A (*pmt1A::URA5*) and *pmt4*A (*pmt4A::URA5*) were incubated in YPD at 30°C and 37°C to an OD_600_ of 1, and cells were subsequently analyzed by light microscopy (DIC). B: Serotype A *pmt4*A strain was incubated in YPD at 30°C to an OD_600_ of 1, and cell suspension was analyzed by light microscopy (DIC) after no treatment or 1 min vortexing or sonication.

**Figure 4:**
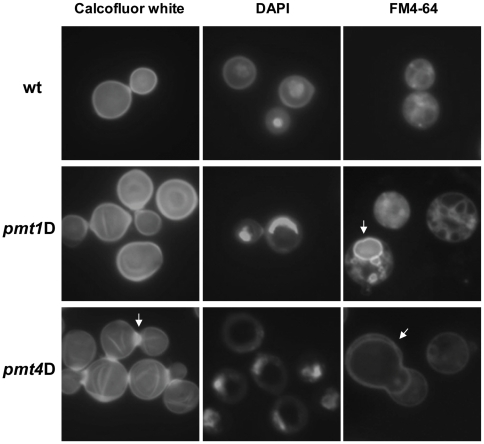
*pmt1* and *pmt4* mutant strains show altered cell wall phenotypes and vacuole distribution. Wild-type serotype A strain H99 and *pmt* mutant strains *pmt1*A (*pmt1A::URA5*) and *pmt4*A (*pmt4A::URA5*) were incubated in YPD at 30°C to an OD_600_ of 1. Cells were fixed for 30 min in 10% formaldehyde and subsequently stained as described with chitin staining dye calcofluor white, the DNA dye DAPI and the vacuolar dye FM4-64. Pictures were taken at 400x using a Zeiss Axioskop 2 Plus Fluorescence Microscope and a AxioCam MRM digital camera.

For other yeasts and filamentous fungi, the loss of certain protein-*O-*mannosyltransferases results in cell morphology defects. In contrast to wild-type cells that grow as single yeasts with simple buds, the *C. neoformans pmt4*A and *pmt4*D mutant strains both exhibited a cell aggregation phenotype when cells were grown for 24 h to 48 h in either YPD or SD medium at 30°C. The cells were often associated in multi-cell aggregates (Figure 3A). In addition, the cell aggregates could not be resolved by vortexing or sonication suggesting that the *Δpmt4* strain may have a defect in cell separation, rather than altered cell adherence (Figure 3B). Calcofluor white staining of cell wall chitin in the *pmt4* mutant strains demonstrated a failure of daughter cells to separate properly from mother cells, with unusually large amounts of cell wall chitin in this area (Figure 4, arrow). These results confirmed recently published data on another *pmt4*A mutant in which cell aggregates were still connected by un-degraded cell wall material as demonstrated by transmission electron microscopy [22]. Moreover, since each single cell retained a single nucleus shown by DAPI staining (Figure 4) there seems to be no obvious effect of the *pmt4* mutation on the cell cycle.

In contrast to the *pmt4* mutant strains, the *pmt1*A and *pmt1*D mutants did not show prominent cell aggregation when grown under the same conditions (Figure 3A), and calcofluor white staining revealed a normal pattern of chitin deposition and cell separation (Figure 4). When incubated at 37°C, the *pmt1* and *pmt4* mutants demonstrated prominent morphological changes compared to wild type. Both mutants grew as large, dysmorphic cells subject to spontaneous lysis. Although DAPI staining revealed single nuclei in all strains, the nuclei in the *pmt1* and *pmt4* mutants were often displaced to the cell periphery by a large central structure. This structure was demonstrated to be an enlarged vacuole by staining with FM4-64 (Figure 4). Although the large vacuole is present in these cells at 30°C, it is most prominent in cells incubated at 37°C. In contrast, wild-type cells displayed multiple, small vacuoles when incubated at similar conditions.

### The *pmt* mutants are sensitive to different cell stresses

**Figure 5:**
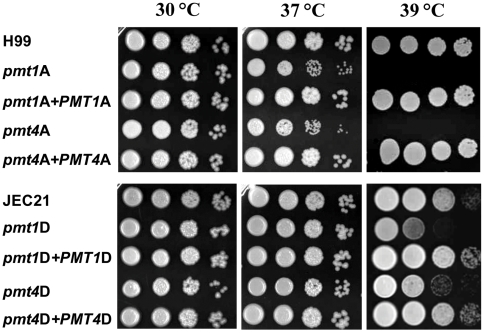
*pmt* mutant strains show growth defects at high growth temperatures. Over-night cultures of wild-type, *pmt* mutant and corresponding reconstituted strains from serotype A (upper panel) and serotype D (lower panel) were adjusted to an OD_600_ of 0.1 and diluted by 10-fold serial dilutions down to a 10^−4^ dilution. 5 µl of each dilution step was spotted onto YPD plates, and plates were incubated at the indicated temperature for 2–3 days. Strains used were the serotype A strains H99 (wild-type), *pmt1*A (*pmt1A::URA5*), *pmt4*A (*pmt4A::URA5*), *pmt1*A*+PMT1*A (*pmt1A::URA5 PMT1A*-*Neo^R^*) and *pmt4*A*+PMT4*A (*pmt4A::URA5 PMT4A*-*Neo^R^*), and the respective serotype D strains JEC21 (wild-type), *pmt1*D (*pmt1D::URA5*), *pmt4*D (*pmt4D::URA5*), *pmt1*D*+PMT1*D (*pmt1D::URA5 PMT1D*-*Neo^R^*) and *pmt4*D*+PMT4*D (*pmt4D::URA5 PMT4D*-*Neo^R^*).

Pmt proteins play important roles in cell wall architecture, and mutations in these proteins often result in increased sensitivity to various cell stresses such as elevated growth temperature, osmotic stress or cell wall destabilising agents. Consistent with the temperature-dependent cell morphology changes in the *pmt* mutants, both *pmt1*A and *pmt4*A strains displayed a marked defect in growth at elevated temperatures (Figure 5). When incubated on YPD medium at 30°C, both mutant strains grew similar to wild type. However, at 37°C, both *pmt* mutants displayed reduced colony size, consistent with a profound temperature-sensitive growth defect (Figure 5). Formal growth curves, measuring changes in the optical density of log-phase cultures in a liquid YPD, confirmed the observations on solid media (data not shown). In contrast to the wild type and reconstituted strains, neither mutant strain grew at 39°C (Figure 5). The *pmt1*D and *pmt4*D mutant strains displayed a similar but less severe temperature-dependent growth defect.

**Figure 6:**
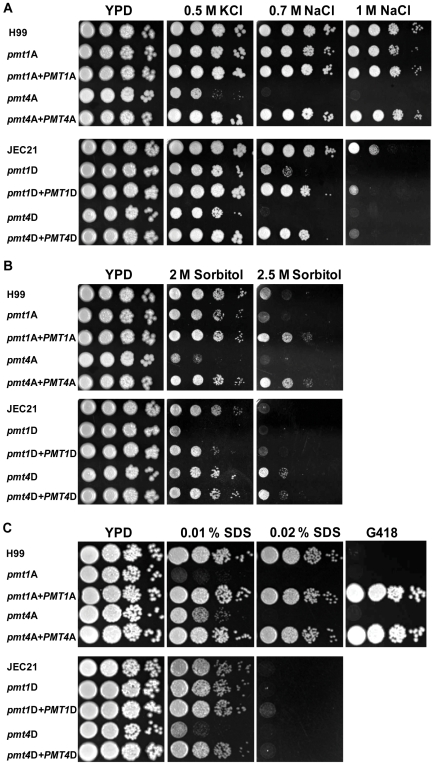
*pmt* mutant strains are sensitive to hyper-osmotic stresses. Over-night cultures of wild-type, *pmt* mutant and corresponding reconstituted strains from serotype A (upper panel) and serotype D (lower panel) were adjusted to an OD_600_ of 0.1 and diluted by 10-fold serial dilutions down to a 10^−4^ dilution. 5 µl of each dilution step was spotted onto YPD plates containing stress agents, and plates were incubated at the indicated temperature for 2–3 days. Strains used were the serotype A strains H99 (wild-type), *pmt1*A (*pmt1A::URA5*), *pmt4*A (*pmt4A::URA5*), *pmt1*A*+PMT1*A (*pmt1A::URA5 PMT1A*-*Neo^R^*) and *pmt4*A*+PMT4*A (*pmt4A::URA5 PMT4A*-*Neo^R^*), and the respective serotype D strains JEC21 (wild-type), *pmt1*D (*pmt1D::URA5*), *pmt4*D (*pmt4D::URA5*), *pmt1*D*+PMT1*D (*pmt1D::URA5 PMT1D*-*Neo^R^*) and *pmt4*D*+PMT4*D (*pmt4D::URA5 PMT4D*-*Neo^R^*). Strains were spotted onto YPD plates supplemented with various salts (A), sorbitol (B) or SDS (C) at indicated concentrations. To test G418 resistance (20 µg/ml) (C), over night cultures were initially diluted to an OD_600_ of 1.0 versus 0.1 used in all other experiments. Plates were incubated at 30°C for 2–3 days.

The *pmt* mutant strains of both serotypes also displayed increased susceptibility to salt stress (Figure 6 A). When incubated in the presence of 0.5 M KCl, the *pmt4*A and *pmt4*D strains grew slower than wild-type/reconstituted strains. Both *pmt4* mutant strains also demonstrated striking growth inhibition by the addition of 0.7 M or 1 M NaCl to the medium (Figure 6A). The *pmt1*A and *pmt1*D strains were also inhibited by NaCl, but to a lesser extent than the *pmt4* strains. The cell wall destabilising agents Congo red, caffeine, and calcofluor white had no significant effect on the growth of the *pmt* mutants in either variety (data not shown).

In contrast to the concordant effects of salt and temperature on the various *pmt* mutants in the two different varieties, the *pmt1* and *pmt4* mutants demonstrated variety-specific differences in the levels of susceptibility to other osmotic and cell wall stress. The *pmt4*A mutant is more susceptible than the corresponding *pmt1*A strain to sorbitol (2 M and 2.5 M) (Figure 6B), and the *pmt1*A mutant is more susceptible to the effects of 0.1% SDS (Figure 6C). In contrast, the *pmt1*D mutant grows very poorly in the presence of sorbitol (Figure 6B), but its growth is unaffected by SDS (Figure 6C). The *pmt4*D strain is not inhibited by high sorbitol concentrations, in contrast to the corresponding *pmt4*A strain; however, the growth of *pmt4*D is inhibited by SDS. Therefore, sorbitol and SDS have very different cell surface destabilizing effects on the *pmt* mutants in the two *C. neoformans* varieties, suggesting that the Pmt proteins play distinct roles in these related but divergent strain backgrounds.

### Virulence factors of *C. neoformans* are differentially affected by the *pmt* mutations

Besides its ability to grow at 37°C, virulence of the fungal pathogen *C. neoformans* is determined by a set of specific virulence factors, including the secretion of various hydrolytic enzymes, the ability to produce a polysaccharide capsule, and the expression of the antioxidant melanin pigment. A common feature of these factors is the involvement of extracellular components. Since protein glycosylation mainly affects extracellular or surface-exposed proteins, we hypothesized that some of these virulence factors would be affected by mutations in genes affecting protein-*O-*mannosylation. Compared to wild type, the *pmt1* and *pmt4* mutants demonstrated no changes in the activity of the secreted enzymes urease or phospholipase B (data not shown), both of which have been linked to virulence of *C. neoformans*.

**Figure 7:**
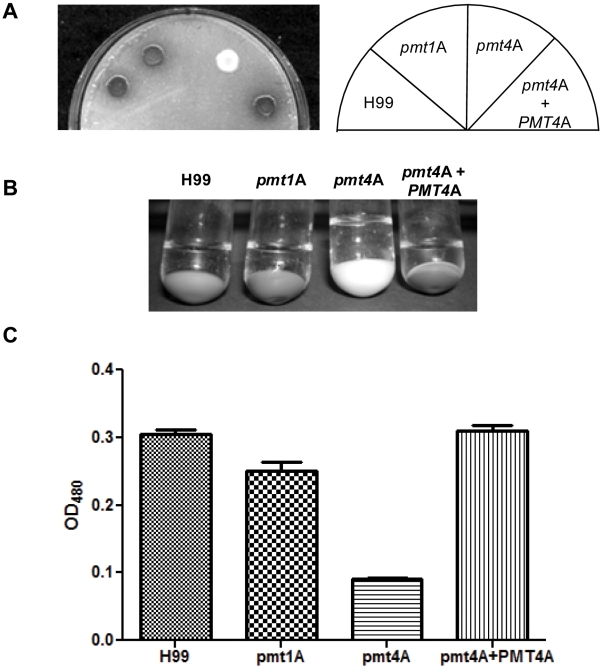
*pmt4* but not *pmt1* mutant strains are delayed in melanin synthesis. A: 10 µl of over-night cultures from serotype A strains H99 (wild-type), *pmt1*A (*pmt1A::URA5*), *pmt4*A (*pmt4A::URA5*) and *pmt4*A*+PMT4*A (*pmt4A::URA5 PMT4A*-*Neo^R^*) were spotted onto standard Niger seed-plates, and plates were incubated for three days at 30°C. B: Cells from 2 ml of the over-night cultures from A were harvested and resuspended in 2 ml glucose-free asparagine-medium supplemented with L-DOPA, and cultures were shaken at 30°C over night. Subsequently cells were pelleted, and cell pellet and supernatant were photographed. C: OD_480_ of the supernatants from cultures in B were determined and graphically displayed using Prism 5 (GraphPad, San Diego, Calif.). Graph shows the overall result of several independent experiments.

Another secreted protein that is essential for virulence of *C. neoformans* is the enzyme laccase, a phenoloxidase that catalyses the rate-limiting step of melanin production. While *pmt1* mutant strains did not show any defect in melanin production, our *pmt4* mutants (both serotype A and serotype D) were delayed in melanin production. The melanin production delay was complemented in the *pmt4+PMT4* reconstituted strain (Figure 7). This finding contrasts with the prior observation of melanin production in a serotype A *pmt4* mutant [22]. This difference may be due to small differences of the disruption constructs used in both studies (see Discussion).

Another virulence-associated phenotype of *C. neoformans* is its ability to produce a large polysaccharide capsule. Using a standard India ink counter-stain, we assessed capsule formation in each of our *pmt* mutant strains. Microscopic analyses of the various serotype A and D mutant strains revealed that all strains made capsule when incubated in DMEM capsule-inducing medium. To control for cell size variation between the mutant and wild-type cells, we quantified relative capsule size by determining the total cell diameter of cells with the surrounding capsule compared to the diameter of the cell itself [32]. The average capsule ratio for 100 wild-type cells was 1.8 (+/−0.02), and this ratio was similar in the *pmt1* mutant 1.75+/−0.05). In contrast, the capsule ratio for the pmt4 mutant was 1.45+/−0.03, indicative of a slightly reduced capsule size (data not shown). Results were identical for serotype A and serotype D strains, and the reduction in capsule size of the *pmt4* strains was complemented by the wild-type *PMT4* allele.

### Protein-*O-*mannosylation and mating

**Figure 8:**
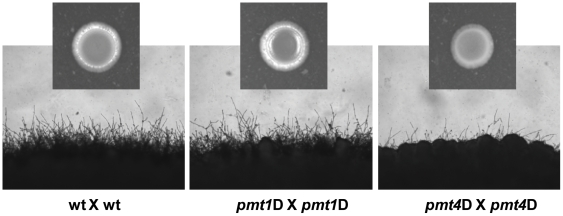
*pmt4*D mutant strains show serotype-dependent filamentation defects. To perform matings of wild-type and *pmt* mutant strains, the respective strains were first grown over-night in YPD at 30°C. Cells were harvested, washed twice with sterile 0.9% saline, and incubated in saline on a shaker for one hour at 30°C. 10 µl of respective strains were mixed, and the cells were spotted onto standard solid V8 agar plates, which were subsequently incubated at RT in the dark. Edges from the colonies and colonies themselves (small pictures) were photographed after 48 h. Shown are bilateral matings of serotype D strains JEC20 and JEC21 (wild-type), SW5 (*MATa*) and SW6 (*MATα*) (*pmt1*D mutants), and SW8 (*MATa*) and SW9 (*MATα*) (*pmt4*D mutants), respectively.

The effect of Pmt's on mating was analyzed in the serotype D strains since these crosses are more vigorous than in serotype A, and since mating type has been associated with virulence in this strain background [33], [34]. The *pmt1* mutant strains did not show any obvious defect in any unilateral (wild-type x *pmt1*) or bilateral (*pmt1* x *pmt1*) crosses. In contrast, the *pmt4* mutation showed notable effects on mating. Unilateral matings of the *pmt4* strains to wild-type testers behaved similar to wild-type control crosses (data not shown). On the other hand, a bilateral cross of two *pmt4* strains revealed a delayed mating reaction, with reduced filament formation after 48 h in comparison to wild-type controls (Figure 8). In addition, less aerial hyphae were produced during this cross, as indicated by the lack of the white mycelial rim surrounding the mating patch (Figure 8; small picture). Microscopic analysis of the hyphal structures produced in a bilateral *pmt4* mating reaction showed irregularly shaped and thickened filaments with swollen distal tips, phenotypes reminiscent of mutations in the *swoA* locus of *Aspergillus nidulans*, a gene that interestingly has recently been found to encode one of three *PMT* genes present in *A. nidulans* [35].

### *pmt* mutant strains are severely attenuated for virulence

**Figure 9:**
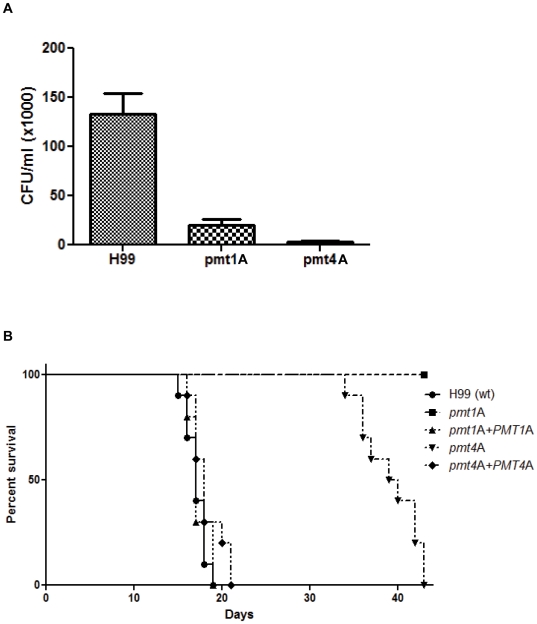
*pmt* mutant strains of serotype A are attenuated for virulence. A: A macrophage killing assay was performed for the serotype A wild-type strain H99 and the *pmt* mutant strains SW13 (*pmt1A::URA5*) and SW14 (*pmt4A::URA5*) as previously described. CFU from two independent experiments with four repetitions of each strain are shown. B: A murine inhalation model was performed for serotype A strains H99 (wild-type), *pmt1*A (*pmt1A::URA5*), *pmt4*A (*pmt4A::URA5*), *pmt1*A*+PMT1*A (*pmt1A::URA5 PMT1A*-*Neo^R^*) and *pmt4*A*+PMT4*A (*pmt4A::URA5 PMT4A*-*Neo^R^*) as previously described. Briefly, for each strain 10 A/Jcr mice were infected intranasally with 10^5^ CFU, and survival time post infection was determined.

Considering the effect of the different *pmt* mutations on growth and virulence-associated phenotypes, it seemed likely that these mutations would also have a negative effect on virulence of *C. neoformans*. We therefore tested the pathogenicity of the serotype A strains in isolated macrophages and in whole animals. When co-incubated with activated J774A.1 macrophages, the *pmt1*A and *pmt4*A strains demonstrated reduced survival at 24 hours compared to wild-type (6-fold and 40-fold reduction, respectively) (Figure 9). These relative differences in macrophage survival are likely due to differences in high-temperature growth and virulence factor expression.

In a murine model of inhaled cryptococcosis, both mutant strains were also significantly reduced in virulence. In this model, 10^5^ *C. neoformans* colony-forming units are intranasally inoculated into complement-defective A/Jcr mice, simulating the natural respiratory route of infection in humans. Animals infected with either wild-type or reconstituted strains had a median survival of 18–19 days, and no mice survived past 21 days after infection. The median survival of mice infected with the *pmt4*A mutant was 40 days (p<0.001 compared to wild-type), and no mice infected with the *pmt1*A strain died from the infection during the course of the 45-day experiment (Figure 9).

## Discussion

Over the past few years, the Pmt-mediated process of protein-*O-*glycosylation has been defined in several species. Pmt's primarily modify proteins targeted for secretion, and this process is essential in most fungi [3], [28], [36]. Its role in fungal pathogenesis has also been explored in *C. albicans* and *C. neoformans* [22], [37]. In this report, we extend prior studies of Pmt function in the human pathogenic yeast *C. neoformans*. First, we demonstrated that *C. neoformans* contains three *PMT* genes, and that each gene encodes one Pmt enzyme for each of the major classes of these proteins. In contrast, other fungi such as *S. cerevisiae* have undergone paralogous duplication of *PMT* genes within these classes, perhaps developing novel functions for various Pmt proteins.

In addition to a conservation of sequence similarity in *PMT* genes, we also demonstrated that the basic function of *C. neoformans* Pmt enzymes is likely similarly conserved. For example, current models of Pmt enzyme activity suggest that the Pmt holoenzymes function optimally as heterodimers [28], [30], [38], and the activity of the Pmt2 class of proteins is a required component of these protein complexes [15], [28]. Consistent with this working model developed in other fungi, we demonstrated that the *PMT2* gene is essential for viability in *C. neoformans*. Additionally, the simultaneous mutation of the *C. neoformans PMT1* and *PMT4* genes seems to be synthetically lethal, as predicted from a model in which Pmt2 must function in concert with other Pmt proteins. Such functional conservation suggests that strategies that block fungal Pmt function might inhibit the growth of diverse fungal species.

Besides being an essential process, we also demonstrated that protein-*O-*glycosylation has a significant effect on the virulence of *C. neoformans*, even in instances in which it is not required for viability. We demonstrated that mutations in either the *PMT1* or *PMT4* genes result in dramatic attenuation in virulence in both a macrophage killing assay and a mouse inhalation model of cryptococcosis, two models that assess different aspects of cryptococcal pathogenesis. Our studies in the role of Pmt4 on *C. neoformans* pathogenesis are consistent with prior reports [22]. However, these experiments demonstrate that the *pmt1* mutant is even more attenuated for survival *in vivo* compared to *pmt4* mutants, despite similar *in vitro* temperature- and cell wall-sensitive phenotypes. Interestingly, both *pmt1* and *pmt4* mutants show high temperature sensitivity at 39°C, which may have a great impact on infectivity in the mouse model. To investigate the role of temperature sensitivity in the virulence attenuation we will test the virulence of these mutants in a heterologous host model, such as wax moths (*Galleria mellonella*), which does not require high temperature during infection.

Virulence of the basidiomycete *C. neoformans* has been linked to several well-defined phenotypes, including the production and export of extracellular factors such as melanin, capsule, and various lytic enzymes [39]–[43]. Of these classic virulence-associated phenotypes, only melanin production was altered in one of these strains, the *pmt4* mutant. *C. neoformans* strains that are defective in melanin production are attenuated for virulence, including strains with mutations in the laccase gene *LAC1*, encoding the rate limiting enzyme of melanin production [39], [40]. Therefore, the delay in melanin production likely plays a role in the reduced virulence of the *pmt4* mutant strain.

The *C. neoformans* laccase Lac1 may also be a direct target of Pmt4. Lac1 is an *N*-glycosylated, cell wall associated protein [44], and *N*-glycosylation often precedes *O-*glycosylation. Altered Pmt4 activity might therefore lead to a mislocalization of Lac1 and a dramatic reduction of melanin production. Our observation of reduced melanin in the serotype A and D *pmt4* mutants is different from a prior report in which no melanin defect was observed in a serotype A *pmt4* mutant [22]. One difference between the two *pmt4* mutants was the method of gene mutation. Between the 5^th^ and 6^th^ transmembrane domains, Pmt proteins are characterized by an extensive loop 5 that is essential for mannosyl-transferase activity [27]. Loop 5 contains three conserved motifs (A–C) that are important for enzyme activity, and domain C is the most C-terminal domain of this region of the protein. Olson *et al.* disrupted Pmt4 downstream of motif C; in contrast, our *pmt4* mutations resulted at least in deletion of domain C in loop 5, supposedly resulting in complete inactivation of the Pmt4 enzyme.

In addition to the melanin delay, the *pmt1* and *pmt4* mutant cells display aberrant cell morphology and pronounced cell aggregation. These morphological changes may be linked to the cell wall- and temperature-sensitive phenotypes identified *in vitro*, resulting in cell lysis under stress. Similarly, hyperflocculant and dysmorphic *C. neoformans* cells demonstrated reduced virulence in a mouse model, as well as increased susceptibility to complement-activated phagocytosis by macrophages [45].

The temperature sensitivity of the *pmt1* and *pmt4* mutants likely explains much of the altered virulence in these strains. Other *C. neoformans* strains with altered growth at mammalian physiological temperatures display similar, predictable virulence defects [46]. Interestingly, in serotype A strains, but not in serotype D, the *pmt1* mutant showed a significant growth defect at 37°C in liquid medium but not on solid medium. The differential growth effect in liquid medium may be caused by higher shearing forces that are not present during growth on a solid medium.

Moreover, previous reports demonstrated that *pmt4* deficient strains showed dramatic differences in the overall pattern of mannosylated proteins by 2D gel electrophoresis [22]. Extracellular mannoproteins are important regarding immunological aspects of *C. neoformans*-host interactions including T-cell activation [47]–[49]. Therefore, defects in protein-*O-*glycosylation may also impair virulence of *C. neoformans* by altering the immunological response to the microbial cells and thereby affect pathogenesis. It therefore will be very interesting to analyze whether the *pmt1* mutation results in similar differences in cell wall mannoprotein composition and which proteins are specifically affected by the *pmt1* and *pmt4* mutation. The identification of immediate targets for individual cryptococcal Pmt proteins may provide further explanations for the defects in pathogenicity found for the *pmt1* and *pmt4* mutants of *C. neoformans*.

Targets of the Pmt enzyme complex have been defined in various fungal species. In fact, the Fks1 protein may be modified by *C. neoformans* Pmt4 [22]. Since Fks1 plays a major role in the synthesis of glucans, which are important components of fungal cell walls, this observation offers a potential mechanism for the cell wall defects described for the *pmt4* mutant strain [22]. Given the similar but distinct phenotypes of the *pmt1* and *pmt4* mutants, it is likely that these two Pmt proteins possess different affinities for Pmt target proteins.

Given the role that protein-*O-*glycosylation plays in microbial pathogenesis, investigators have explored Pmt proteins as potential targets for therapeutic intervention. Derivatives of rhodanine-3-acetic acid have recently been identified that specifically inhibit the Pmt1 isoform in *C. albicans*. Treating wild-type cells with these inhibitors resulted in phenotypic and transcriptional changes reminiscent of *C. albicans pmt1* deletion strains [15], [50], [51]. Since Pmt2 and Pmt4 orthologs are present in mammals, the possibility of developing Pmt isoform-specific inhibitors renders Pmt proteins an attractive target for antifungal drug development. Additionally, targeting specific Pmt isoforms with inhibitory compounds may also be useful in killing phytopathogenic fungi, since Pmt proteins have yet to be identified in plants [3].

The cell wall defect in the *pmt1* and *pmt4* mutants is striking. One mechanism proposed for the Pmt4 control of *C. neoformans* cell wall integrity was recently provided. The stress related induction of the Fks1 gene, encoding the catalytic subunit of β-1,3-glucan synthase, is dependent on Pmt4 [22]. Since β-1,3-glucan is a major component of fungal cell walls, altered glucan synthesis would likely result in altered cell integrity under stress conditions [52]. Moreover, defects in *C. neoformans* PKC/cell integrity signalling resulted in phenotypes similar to those in the *pmt* mutant strains [53]. These phenotypes included morphological defects, defects in vacuolar biogenesis, growth defects at elevated growth temperatures or low SDS concentrations, and a higher sensitivity to osmotic stresses. Even more interesting it has been shown that proper PKC/cell integrity signalling is necessary for melanin production; *pkc1* mutants show improper laccase localization and reduced melanin production. The cell wall abnormalities, and resulting changes in PKC signalling, may also explain the melanin production delay of the *pmt4* mutants.

Levitz and Specht identified 55 potential GPI anchored membrane proteins with serine/threonine rich regions that are thought to be potential *O-*glycosylation targets [54]. Within these proteins there were several candidates that may be involved in remodelling the cell wall including three chitin-deacetylases (Cda1-3) and three potential endoglucanases (AAW45003, AAW46063 and AAW46065). It will be interesting to see whether any of these proteins will be identified as a direct target of Pmt-dependent *O-*glycosylation and if deletions of any of the corresponding genes will result in morphological phenotypes similar to the *pmt* mutants.

In conclusion, the *PMT* gene encodes a group of proteins critical in the biology and virulence of *C. neoformans.* Given the high degree of conservation in this gene family in other pathogenic fungi, this gene family is an ideal antifungal drug target. Future studies should explore this possibility and further define the mechanisms behind the *PMT* family mutant defects.

## Materials and Methods

### Strains and media

Reference strains used in this study where the congenic serotype D strains JEC20 (*MATa*) and JEC21 (*MATα*), and the serotype A strain H99. *pmt* mutant strains where constructed transforming the JEC20/21 derivatives JEC155 (*MATα*, *ade2 ura5*), JEC156 (*MATa*, *ade2 ura5*), or F99, respectively, a 5-FOA resistant derivative of H99. All strains used in this study are listed in Table 1. Yeast-peptone-dextrose (YPD) and yeast nitrogen base media, synthetic (SD) medium, V8 agar for mating, filament agar, Niger seed for melanin production, and serum-free Dulbecco's modified Eagle's medium for capsule induction were prepared as previously reported [32], [53], [55], [56].

**Table 1:** Strains used in this study.

**Serotype A strains**	**Genotype**	**Source/reference**
H99	wild-type *MATα*	[67]
F99	*MATα ura5* (5-FOA resistent isolate of H99)	Ping Wang (Heitman-laboratory)
SW13	*MATα pmt1::URA5*	this study
SW14	*MATα pmt4::URA5*	this study
SW15	*MATα pmt1::URA5 PMT1-Neo^R^*	this study
SW16	*MATα pmt4::URA5 PMT4-Neo^R^*	this study
**Serotype D strain**	**Genotype**	**Source/reference**
JEC20	wild-type *MATa*	[68]
JEC21	wild-type *MATα*	[68]
JEC155	*MATα ade2-27 ura5*	Jeff Edman
JEC156	*MATa ade2-27 ura5*	Jeff Edman
JEC157	*MATα ade2 ura5 lys1*	Jeff Edman
JEC34	*MATa ura5*	Jeff Edman
JEC43	*MATα ura5*	Jeff Edman
JEC50	*MAT*α *ade2-27*	Jeff Edman
SW1	*MATα pmt1::ADE2*	this study
SW2	*MATa pmt1::ADE2*	this study
SW4	*MATa pmt1::URA5 ade2-27*	this study
SW5	*MATa pmt1::URA5*	this study
SW6	*MATα pmt1::URA5*	this study
SW7	*MATa pmt4::ADE2 ura5*	this study
SW8	*MATa pmt4::ADE2*	this study
SW9	*MATα pmt4::ADE2*	this study
SW10	*MATα pmt4::URA5 ade2-27*	this study
SW11	*MATa pmt4::URA5*	this study
SW12	*MATα pmt4::URA5*	this study
SW17	*MATα pmt1::URA5 PMT1-Neo^R^*	this study
SW18	*MATα pmt4::URA5 PMT4-Neo^R^*	this study
SW19	*MATα pmt4::ADE2 ura5*	this study

### Plasmid construction

*PMT* homologs were identified by tblastn searches using *S. cerevisiae* Pmt1 and *C. albicans* Pmt1 protein sequences against the National Center for Biotechnology Information (http://www.ncbi.nlm.nih.gov). For generating *PMT* disruption constructs *PMT* open reading frames (ORF) were amplified by PCR from reference strains H99 and JEC21, and fragments obtained were cloned into standard cloning vectors. Except for *PMT1*A *Bgl*II sites were integrated into the *PMT* ORFs using oligonucleotide based site directed mutagenesis (Stratagene Quick Change site directed mutagenesis kit). Subsequently, *ADE2* or *URA5* containing *Bam*HI fragments were cloned into the *Bgl*II sites to disrupt the respective ORFs after amino acid E468 (2A), K461 (4A), W315 (1D), N508 (2D) and Q412 (4D), respectively. For *PMT1*A an existing *Eco*RV restriction site was used to generate the disruption constructs. In this case *ADE2* and *URA5* containing *Sma*I fragments were used for disrupting the *PMT1*A ORF at position D439. These constructs were then PCR amplified to generate a linear disruption allele and gel purified for use in biolistic transformation into appropriate auxotroph strain [57]. Biolistic transformations were conducted using established methods [58]. For reconstituting the generated disruption strains the *PMT* ORFs plus additional 500–700 bp up- and downstream of the respective START and STOP codons were amplified by PCR with primers containing *Bam*HI restriction sites. Corresponding *Bam*HI fragments were subsequently cloned into the *Bam*HI restriction site of vector pJAF1 containing the Tn5 derived *Neo* ^R^ resistance marker [59].

The primers utilized in plasmid construction are presented in Table S1.

### Strain construction

To generate a homozygous serotype D diploid *ade2* strain, strains JEC156 and JEC157 were crossed according to Sia et al. (2000) and random spores were isolated on SD solid media supplemented with 20 µg/ml adenine. Plates were grown for several days at 37°C to prevent filament formation usually seen with diploid strains. In a second step, reddish Ade^−^ strains were isolated and re-streaked onto the same plates, but incubated at RT to induce filament and basidiospore formation typical of diploid *Cryptococcus* strains under these conditions. To generate a heterozygous *pmt2/PMT2* serotype D strain, a resulting diploid *ade2/ade2* diploid strain was transformed with an *ADE2* based *pmt2*D-disruption construct using biolistic transformation. Heterozygous mutants were initially identified by colony PCR and subsequently confirmed by Southern analysis.

### RNA preparation

Strains were incubated to mid-logarithmic phase at 30°C in YPD medium. Cells were collected by centrifugation and flash frozen on dry ice. Total RNA was extracted from lyophilized cells using the TRIzol reagent (Invitrogen Life Technologies, Carlsbad, CA).

### Real-time PCR

Total RNA was isolated from the relevant strains as described above. The RNA was treated with RNase-free DNase, and cDNA was synthesized using oligo(dT) primers from the SuperScript first-strand synthesis reverse transcription kit (Invitrogen). The resulting cDNA was used as template for quantitative real-time PCR using iQ SYBR Green Supermix (Bio-Rad) according to the manufacturer's specifications. The iCycler iQ multicolor real-time detection system was used as the fluorescence detector with the following PCR conditions: an initial denaturing cycle of 95°C for 3 min and 40 cycles of denaturation at 95°C for 10 s and annealing/extension at 58°C for 330 s. These cycles were followed by a standard melting curve from 53°C to 93°C with fluorescent monitoring each 0.5°C. These data confirmed the amplification of a single product for each primer pair and the lack of primer dimerization. Reactions were performed in triplicate, and the data were expressed as an average cycle threshold, ± one standard deviation. Standard PCRs were run with fivefold dilutions of the cDNA template to determine the optimal amount of template and optimal annealing temperature for the experimental and reference reactions, using 500 nM of each primer.

Gene amplification for each strain and condition was normalized against the constitutively expressed *GPD* gene [60]. Induction (*n*-fold) was calculated relative to the wild-type strain JEC21 using the Bio-Rad iCycler software system, which utilizes the comparative cycle threshold statistical methods as previously described [61]

### Mating analyses and haploid filamentation

To determine defects in mating, the various *pmt* mutant and tester strains were pre-grown on YPD plates at 30°C for 2–3 days, a small amount of growth was removed using a sterile tooth pick and spotted onto solid V8 plates (pH 5.0 or 7.0), either alone or mixed with a respective tester strain. Plates were incubated at room temperature (RT) in the dark for at least 3 days and mating and haploid filamentation was assessed by light microscopy.

### Basidiospore isolation

To isolate basidiospores from genetic crosses, strains were crossed on solid V8 plates as has been described above for the mating analyses and incubated at RT in the dark until basidiospore formation was noticeable. Areas showing basidiospores were excised and single basidiospores were transferred to fresh YPD plates using a micromanipulator (single spore isolation). Alternatively, excised filaments were transferred to a 1.5 ml reaction tube containing 500 µl water, were vortexed vigorously, and spores were subsequently plated on fresh YPD plates or respective selective media (random spore isolation). To isolate haploid Ade2^+^ strains from the heterozygote serotype D *pmt2/PMT2* strain a random spore analysis was done spreading spores on SD plates containing 20 µg/ml adenine, and plates were incubated at 37°C to prevent filamentation. Subsequently, only white colonies were re-streaked onto SD plates that were incubated at RT. Strains that would filament under these conditions were discarded since filamentation would indicate that these strains are still of diploid nature.

### Serial dilution patch tests

Standard serial dilution patch tests were performed as follows: strains to be tested were pre-grown over night in 5 ml liquid YPD medium at 30°C into stationary phase. OD_600_ was determined the next day, cultures were diluted to an OD_600_ of 1, and tenfold serial dilutions were made in YPD down to a dilution of 10^−4^. Finally, 5 µl of the various dilutions were spotted onto the respective solid media and incubated at the indicated temperatures.

### Microscopy

Light, DIC and fluorescence microscopy pictures were taken using a Zeiss Axioskop 2 Plus Fluorescence Microscope mounted with a AxioCam MRM digital camera, or alternatively with a Nikon Eclipse E400 microscope and a Nikon CoolPix 990 digital camera. Cell wall material and genomic DNA were stained using the Fluorescent Brightener 28 (calcofluor white; Sigma Aldrich) or 4,6-diamidin*O-*2-phenylindole (DAPI; Molecular Probes). First, *C. neoformans* strains were grown to an OD_600_ of ∼1, cells were harvested and fixed for 30 min in 10% formaldehyde. Subsequently, cells were washed three times with PBS and then permeabilized with 1% Triton X-100 for 10 min. Finally, cells were once more washed three times with PBS and stained with the indicated dyes. To visualize vacuoles, cells were stained with the lipophilic dye *N*-(3-triethylammoniumpropyl)-4-(6-(4-(diethylamino) phenyl) hexatrienyl) pyridinium dibromide (FM 4–64; T-3166; Invitrogen Corporation, Carlsbad, CA) as described previously [62], [63]. Briefly, logarithmically growing *C. neoformans* cells corresponding to 1 ml of OD_600_ of ∼0.1 were harvested and resuspended in 100 µl YPD. Subsequently, the vacuolar stain FM4-64 was added to a final concentration of 40 µM, and cells were incubated on a rotary shaker for 45 min at 30°C. Before microscopy cells were washed several times with YPD.

### *In vitro* virulence assays

To determine capsule production, strains of interest were grown over night in Dulbecco's modified Eagles medium for 3 days at 30°C [32]. Subsequently, 5 µl of the culture were mixed with an equal volume of India ink, and cells were analyzed by light microscopy. Melanin production was determined by two different techniques. First, 10 µl of over-night cultures were spotted onto standard Niger seed plates, and plates were incubated at 30°C for 2–4 days until the controls showed a brownish colour indicative of melanin production. Second, cells from 25 ml YPD over-night cultures were harvested and resuspended in 2 ml of glucose-free asparagine-medium supplemented with L-DOPA, and cell suspensions were incubated for 24 h at 30°C on a rotary shaker. Subsequently cells were pelleted, and melanin production was documented by photographing the cell pellet, and determining the OD_480_ of the supernatants [64]. Urease activity tests on wild-type and *pmt* mutant strains were performed in the following way. One colony of each strain was added to sterile deionized H_2_O in a microcentrifuge tube and vortexed vigorously. One BBL TAXO™ urease differentiation disk (Becton, Dickinson, & Co., Sparks, MD) was added to each tube using sterile techniques, which then were incubated at 30°C and checked at 10 min and 30 min for urease activity according to manufacturer's instructions.

### *In vivo* virulence analyses

Overall virulence of *C. neoformans* strains was tested by macrophage killing assays and by a murine inhalation model. For the macrophage killing assay 50 µl of freshly grown J77A.1 macrophages (∼1×10^5^ J774A.1 macrophages in DMEM) were pipetted into 96 well microtiter plates and activated by adding 50 µl DMEM supplemented with INF-γ (100 U/ml) and LPS (0.6 µg/ml). Negative controls were “activated” with DMEM containing no additional supplements. Microtiter plates were then incubated for 12–18 h at 37°C under a 5% CO_2_ atmosphere to generate a macrophage cell-layer. *C. neoformans* strains to be tested were grown over night in liquid YPD medium, cells were harvested and washed three times with PBS. Cells were resuspended in DMEM containing 1 µg/ml monoclonal antibody mAb18B7 at a cell titer of 1×10^6^ living cells per ml, determined by Trypan Blue staining, and incubated for 1 h at 37°C at 5% CO_2_. Subsequently, 100 µl *C. neoformans* cells were pipetted on top of the macrophage cell layers, and microtiter plates were incubated at 37°C at 5% CO_2_ for one hour. Non incorporated cryptococcal cells were removed by carefully washing the macrophage cell layers three times with PBS. Finally cell layers were covered with 150 µl fresh DMEM culture medium and incubated for another 24 h at 37°C at 5% CO_2_. To determine survival rates of cryptococcal cells the covering culture medium was removed and transferred to a fresh reaction tube. Macrophages in the titer plates were incubated with 100 µl 0.5% SDS solution for ∼5 min at RT, cells were lysed by pipetting cells up and down several times, and the cell lysate was combined with its respective culture supernatant. Finally, wells were washed once with 200 µl PBS solution, which was subsequently added to the respective cell lysate. Cell lysates were diluted 300 fold, and cryptococcal CFU were determined by standard techniques on solid YPD plates, incubated at 30°C. Macrophage killing assays were repeated at least three times [65]. The murine inhalation model of systemic Cryptococcosis was performed as described before [66]. Briefly, groups of ten female A/Jcr mice (∼20 g body weight) were infected intra-nasally with 10^5^ cryptococcal CFU resuspended in PBS. To determine survival rates, mice were inspected for vitality twice a day, and individuals showing ∼15% weight loss, neurological abnormalities or extreme anorexia were sacrificed immediately according to the animal protection regulations of the Duke University Medical Center Animal Care & Use Program. Mice were maintained at the Research Institute for Children Animal Facility in accordance with the American Association of Accreditation of Laboratory Animal Care guidelines.

### Statistical analysis

The software program Prism 5 (GraphPad, San Diego, Calif.) was used for all statistical tests. Log-rank tests were utilized to determine significance of survival in animal studies.

## Supporting Information

Table S1 Plasmids used in this study
(0.02 MB XLS)
